# Association between team functioning and self-efficacy and quality of life for primary care patients in British Columbia, Nova Scotia, and Ontario

**DOI:** 10.1017/S1463423626100887

**Published:** 2026-02-12

**Authors:** Innocent Ndateba, Farinaz Havaei, Kristen R. Haase, William Hogg, Ruth Martin-Misener, Sabrina T. Wong

**Affiliations:** 1 School of Nursing, University of British Columbia, Vancouver, Canada; 2 Family Medicine, University of Ottawa, Ottawa, Canada; 3 School of Nursing, Dalhousie University, Halifax, NS, Canada

**Keywords:** Multimorbidity and social vulnerability, patient-reported outcome measures, primary health care, team-based care

## Abstract

**Aim:**

We aimed to examine the association between team functioning in primary care and patients’ self-efficacy and quality of life. We also examined the moderation effect of multimorbidity and social vulnerability on this association.

**Background:**

Team-based care has been adopted as an appropriate model to deliver comprehensive primary care services to meet the complex needs of patients. Little is known about the association between team functioning and patients’ self-efficacy for managing chronic conditions (SEMCD) and quality of life.

**Methods:**

We used mixed-random effect modelling to analyse secondary cross-sectional data. Data were collected in primary care practices in three Canadian regions. Dependent variables included patients’ SEMCD and quality of life. The independent variable was team functioning measured using the Team Climate Inventory scale (TCI). We also included two interaction terms: social vulnerability and TCI, and multimorbidity and TCI. Control variables included patient characteristics, patients’ experience with care and practice characteristics.

**Findings:**

Eighty-seven practices and 1,929 patients participated in the study. Of these, 67% were female, 5% had two or more social vulnerabilities and 65% had multimorbidity. Regression analyses failed to find an association between team functioning and patients’ self-efficacy or quality of life. There was a strong positive association between team functioning and self-efficacy for people with multimorbidity (*p* = .005) compared to those without multimorbidity. There was also a strong positive association between team functioning and quality of life for those with two or more vulnerabilities (*p* < .001) but not for those with fewer vulnerabilities. The findings showed people with multimorbidity and increased vulnerabilities could benefit from well-functioning teams. Supporting better team functioning through effective communication (e.g., team meetings) and care coordination; encouraging full participation of all team members in service delivery; and establishing clear team objectives, roles and responsibilities can better meet the needs of complex patients.

## Introduction

Primary care reforms place an emphasis on access to care, better meeting the health care needs of those with access difficulties (Health Canada, 2007; Donnelly *et al.*, 2023; Mitra *et al.*, 2025), and care for complex patients (MacPhee *et al.*, 2020). Team-based primary care (herein referred to as TBPC) is proposed to meet both these objectives (Shi *et al.*, 2017; Szafran *et al.*, 2018; Mitchell *et al.*, 2019; Somé *et al.*, 2020). In TBPC, professionals from different disciplines work collaboratively to provide patient-centred and coordinated primary care services (American Nurses Association, 2016; Wagner *et al.*, 2017; Frogner *et al.*, 2018). Members of teams are interdisciplinary and can include physicians, nurse practitioners, registered nurses, social workers, pharmacists, dietitians, physiotherapists and others (Wagner *et al.*, 2017; Mitra *et al.*, 2025). These professionals can use and exchange their knowledge, skills, and discipline-specific competencies to complement each other and provide holistic, appropriate care to patients.

For complex patients such as people living with multimorbidity (PLWMM) – two or more chronic conditions in one person (Fortin *et al.*, 2012), evidence overwhelmingly shows that TBPC is effective. For example, current systematic reviews indicate that TBPC has positive effect on improved mental health – reduced depression symptoms and anxiety, and patient-centred care (Li *et al*., 2023), disease management and general health outcomes, particularly self-efficacy for managing chronic conditions (SEMCD) and quality of life, medication management, psychosocial and physical functioning, and self-rated health (Ndateba *et al.*, 2025). Other studies indicate reduced health services utilization and care cost as result of TBPC (Levis-Peralta *et al.*, 2020; Mateo-Abad *et al.*, 2020).

Despite the overall positive impact of TBPC, some individual studies failed to find a positive association between TBPC and patient outcomes (Bates *et al.*, 2025; Li *et al*., 2023; Mateo-Abad *et al*.,^2020^). Thus, the exact mechanisms by which TBPC can improve these outcomes remains unknown. Without understanding potential mechanisms, it is challenging to implement best strategies for increasing and sustaining high functioning TBPC. Past work suggests the effectiveness of TBPC heavily relies on team functioning (Salas *et al.*, 2015; Rosen *et al.*, 2018; Darcis *et al.*, 2025). Team functioning is defined as processes and interpersonal interactions that happen within or between team members when executing their tasks to achieve common goals (Cooke and Hilton, 2015). Effective team functioning is characterised by clear roles and responsibilities, good communication, shared vision and mission, mutual trust and respect, and creativity (Mitchell *et al.*, 2012; Gocan *et al.*, 2014). When teams are well-functioning, primary care teams are enabled to adequately provide support to patients (Mateo-Abad *et al.*, 2020), enhance care coordination and communication as well as task delegation between care team members (Altschuler *et al.*, 2012; Bodenheimer and Smith, 2013; Frogner *et al.*, 2018). Effective team functioning enhances teams’ ability to work efficiently (Rotenstein *et al*., 2024). For example, teams with good coordination and communication can potentially prevent duplications of treatments, diagnostic tests, multiple appointments and conflicting advice that people with chronic conditions may receive from multiple professionals regarding their chronic condition management (Gobeil-Lavoie *et al.*, 2019; Kvarnström *et al.*, 2021).

Moreover, effective team functioning has the potential to improve patient outcomes in PLWMM and/or social vulnerabilities such as lower socioeconomic status, lower social support, substance use and mental health disorders (Meyers *et al.*, 2019; Schuttner and Parchman, 2019; Haggerty *et al.*, 2020; Geda *et al.*, 2021; Sum *et al.*, 2021; Deville-Stoetzel *et al.*, 2023).

These individuals may highly benefit from effective team functioning because it can help address the coordination challenges and barriers to healthcare access they often face, while also ensuring the provision of holistic health care services. There still remains gaps in our knowledge of team functioning. There is limited attention to understanding the influence of team functioning on people with multimorbidity (Orueta *et al.*, 2014; WHO, 2016; Poitras *et al.*, 2018) and social vulnerabilities, even though it is known that these patients experience poorer health outcomes such as quality of life (Makovski *et al.*, 2019; Jiao *et al.*, 2020; Foster and Niedzwiedz, 2021; Nwadiugwu, 2021) and challenges in managing their chronic conditions (Al-Hanawi, 2021; Fracso *et al.*, 2022; Osokpo *et al.*, 2022). Without such attention and an equity-focused approach, reforms intended to strengthen primary care may inadvertently reinforce – rather than mitigate – existing health inequities.

Further, self-efficacy – a significant factor for patient self-management (Gobeil-Lavoie *et al.*, 2019) and quality of life (Peters *et al.*, 2019), and quality of life outcomes are rarely studied in the research examining the association between team functioning and primary care patients. One study (Becker and Roblin, 2008) found a positive association between team functioning and patient’s activation—a person’s knowledge, skills and confidence of managing health condition (s) and self-care ability (Sum *et al.*, 2021). Little is known about the association between team functioning and primary care patients’ SEMCD and quality of life accounting for multimorbidity and social vulnerabilities. The purpose of this study was to examine the association between team functioning and SEMCD and quality of life among primary care patients in Canada using intersectional approach. Specifically, we asked:

1. What is the association between team functioning and self-efficacy and quality of life among primary care patients?
2. Do social vulnerability and multimorbidity moderate the association between team functioning and self-efficacy and quality of life among primary care patients?

## Methods

Our analysis is informed by Crenshaw’s intersectionality approach (Crenshaw, 1989). Intersectionality theory posits that social identities (e.g., age, gender, race, socioeconomic status, immigration) and social structures of power influence how individuals experience life (Bauer *et al*., 2021). These social identities interact with structural systems of power (e.g., ideology, economic, political and cultural factors) to jointly promote, address or reinforce inequities (Strauss and Brown, 2021). It is understood that structural power within the primary care system such as organization of primary care delivery (e.g., team-based care model) and how primary care teams function intersect with social identities to influence experiences of individuals about health care services delivery and how these services meet or do not meet their health care or social needs, subsequently impacting health outcomes (Strauss and Brown, 2021). Intersectionality offers a valuable lens for examining whether the team functioning in primary care benefit not only affluent patients but also those with complex health care needs or social vulnerabilities.

**Study design and aim.** We used secondary data analysis of the TRANSFORMATION study (Martin-Misener *et al*., 2019), which employed a cross-sectional survey design to collect data from primary care practices and their patients.

### Settings and participants

TRANSFORMATION study was conducted in Fraser East, British Columbia (BC); Eastern Ontario Health Unit, Ontario (ON); and Central Zone, Nova Scotia (NS) in 2014–2016 (Martin-Misener *et al.,* 2019). Detailed methods about this study can be found elsewhere (Wong *et al.*, 2018; Martin-Misener *et al.,* 2019). Briefly, staff and patients in primary care practices, herein practices, participated in the study. Practices completed a survey about practice characteristics; staff completed the Team Climate Inventory (TCI) and patients completed a survey about their experiences and outcomes. A research assistant consecutively recruited patients in the practice waiting room on days agreeable with each practice. Staff and patients completed surveys, respectively. The study was approved by Institutional Review Boards of Fraser Health, the University of British Columbia, the Ottawa Health Science Network, Bruyère Continuing Care, and the Nova Scotia Health Authority. Written informed consent was obtained from each practice and participating patients. First, researchers recruited practices. Then, patients within each practice were recruited in a consecutive manner over a period of a week.

### Measures

#### Outcome 1: self-efficacy for managing chronic conditions (SEMCD)

This six-item SEMCD scale is a measure of patients’ confidence in their ability to manage their chronic conditions (Lorig and Holman, 2003). The responses are based on a 10-point Likert scale ranging from 1 (not confident at all) to 10 (totally confident). The SEMCD score was constructed by averaging the scores of all six items, with a higher score indicating higher self-efficacy. Previous research supports its validity (Chow and Wong, 2014; Ritter and Lorig, 2014; Nia *et al.*, 2019) and reliability (Tavakol and Dennick, 2011; Paiva *et al.*, 2014; Ritter and Lorig, 2014). In this study, the SEMCD had an acceptable Cronbach’s alpha of 0.90.

#### Outcome 2: quality of life (EQ-5D-5L)

The EQ-5D-5L measures five dimensions of quality of life including mobility, self-care, usual activity, pain/discomfort and anxiety/depression with five levels of severity for each dimension (Oemar and Janssen, 2013). The EQ-5D-5L is a widely used instrument (McCaffrey *et al.*, 2016) with evidence supporting its validity (Conner-Spady *et al.*, 2015; Li *et al.*, 2019; Feng *et al.*, 2021), and reliability (Tran *et al.*, 2012; Seng *et al.*, 2020). In this study, the EQ-5D-5L had an acceptable Cronbach’s alpha of 0.80. We created the Health Utility Index [HUI](value associated with health state profile) from the EQ-5D-5L using the value set — a method to numerically describe and value health-related quality of life, ranging from 1 for ‘perfect health’ and 0 for dead, whereas negative value for health state worse than dead (Xie *et al.*, 2016; Sullivan *et al.*, 2021). We used the value set for Canada (the worst for EQ-5D-5L state of 55555 = −0.148 and the best EQ-5D-5L state of 11111 =: 0.949) to determine a HUI for participants (Xie *et al.*, 2016). A higher HUI indicates better quality of life.

### Independent variable: team functioning

The TCI is a 19-item instrument used to measure team functioning (Beaulieu *et al.*, 2014). The TCI has four dimensions that include (1) Participative safety (six items); (2) Support for innovation and new ideas (five items); (3) Vision or team objectives (four items); and (4) Task orientation (four items). Response options are on various point-Likert scales. Participative safety, support for innovation and new ideas are on five-point scale ranging from 1 (strongly disagree) to 5 (strongly agree). Vision or team objectives is on a seven-point scale ranging from 1 (not at all) to 7 (completely). Task orientation is also on a seven-point scale ranging from 1 (to every little extent) to 7 (to a very great extent) (Beaulieu *et al.*, 2014). The TCI is a valid and reliable measure of team functioning globally (Anderson and West 1998), and was validated in both French and English in Canada (Beaulieu *et al.*, 2014). In this study, the overall TCI score had an acceptable Cronbach’s alpha of 0.94. We constructed overall TCI score by adding up all dimension scores and divided by 4. We calculated TCI scores for only practices with sufficient TCI data – practices with value of ≥ 0.24 (Dawson, 2003) and for individual staff who answered at least 60% of the TCI items. The value was obtained by (N−n)/Nn, where ‘N’ is the total number of staff in the practice and ‘n’ indicates the total number of staff who answered at least 60% of the items of the TCI survey (Dawson, 2003). For example, at least four respondents were needed (TCI data value = 0.17) to retain the practice of 12 staff. We converted overall TCI scores to 0–100 scale to facilitate the interpretation, with a higher score indicating higher team functioning.

### Moderator variables

**Social Vulnerability Index.** Based on previous work, we constructed a social vulnerability index using six items: less than high school, poor/very tight financial status, Indigenous status, speaking another language at home, new immigrant and low social support (Haggerty *et al.*, 2020). The sum of social vulnerabilities ranged from 0 to 6, and was then dichotomized into two categories (1 = 2 or more social vulnerability indicators, 0 = fewer than 2 social vulnerability indicators) (Haggerty *et al.*, 2020).

**Multimorbidity.** Participants self-reported if they had ever been diagnosed with one or more of many chronic conditions (see supplemental Appendix A). There was also an option to specify another condition which was not in the list. We considered multimorbidity for participant with two or more chronic conditions as opposed to 0 or 1 chronic condition (Fortin *et al.*, 2012).

### Control variables

Supplemental Appendix B provides details of control variables. Practice level variables included team size, presence of registered nurses in the clinics and physician’s payment model. Patient characteristics include age, sex, household income, self-rated general health. Additionally, patient experiences with the help from the health care team were also included.

### Statistical analyses

We used descriptive statistics to describe the distribution of sample and bivariate analyses to determine the relationship between independent and outcome variables. We used parametric tests for normally distributed data (e.g., self-efficacy) and non-parametric tests for skewed data (e.g., quality of life) (Polit and Beck, 2017). Given the nested nature of data where patients are nested within practices, we used a multi-level mixed-random effects linear regression models to adjust the association for clustering (Nezlek and Mroziński, 2020).

We investigated our research questions using regression analyses and interaction terms. We performed linear regression for the self-efficacy outcome and generalized linear mixed model (GLMM) using Gamma distribution and Log link function for the quality of life outcome. The GLMMs is a model of mixed-random effects linear regression analysis that is appropriate for skewed data (Lo and Andrews, 2015). Team functioning, individual and practice-level control variables as well as the two interactions terms (e.g., TCI*social vulnerability, and TCI* multimorbidity) were entered in the model simultaneously. We used the mean centred TCI score to plot significant interaction terms between TCI and moderators to facilitate the interpretation and improve sense making of the results (Iacobucci *et al.*, 2016; Goldstein, 2023). We conducted analyses using the Statistical Package for the Social Sciences (SPSS) version 21 (Corporation, 1989) and Jamovi.2.4.8 version to plot significant interactions (The Jamovi Project, 2023). The statistical significance level was set at *p* < .05 (2-sided).

## Results

A total of 87 practices, 566 staff and 1929 patients participated in the study.

### Patient characteristics

Table 1 shows the patient characteristics. The majority (67%) were female with an average age of 53.5 (*SD* = 16.7). About 65% of participants reported having multimorbidity. Almost all spoke English or French and were born in Canada. Approximately, four percent self-identified as Indigenous/Aboriginal. Participants self-identified across a number of Ethno-cultural groups including European descent, South Asia and others (e.g., African, Arab, etc.). Two thirds had completed a high school or technical college diploma. One third reported having poor/very tight financial status while 40% had household income between $15,000 to less than $50,000. Over 90% reported having high social support. Five percent had two and more social vulnerability indicators. Overall, participants rated their general health as good (*M* = 3.0; *SD* = 1.0).

**Table 1.** Patient sociodemographic characteristics, patients’ experiences with help from the health care team, self-efficacy and quality of life (*N* = 1929)

**Variables**	**0 or 1 chronic disease** ***n*** **= 674**	**≥ 2 chronic diseases – Multimorbidity** ***n*** **= 1255**	**Total (*N* = 1929)**
Age, M(SD)***	45.2 (16.4)	58.3 (15.0)	53.7 (16.7)
***Sex, n (%)***			
Female	449 (66.7)	837 (66.9)	1286 (66.8)
***Spoken language at home, n (%)***			
English or French	586 (97.5)	1213 (98.4)	1807 (98.1)
Another language	15 (2.5)	20 (1.6)	35 (1.9)
***Aboriginal status, n (%)***			
Yes	18 (2.7)	56 (4.5)	74 (3.8)
***+Ethno-cultural group, n (%)***			
Caucasian (e.g., European descent)	522 (92.6)	1063 (94.2)	1585 (93.6)
South Asian (e.g., East Indian, Pakistani, Sri Lanka)	20 (3.0)	27 (2.4)	47 (2.8)
Other	35 (5.2)	55 (3.4)	90 (5.4)
Immigrant status, n (%) **			
Not an immigrant	543 (91.0)	1079 (90.1)	1622 (90.4)
Established immigrants (> 10 years)	38 (6.4)	‘108 (9.0)	146 (8.1)
New immigrants (≤10 years)	16 (2.7)	11 (0.9)	27 (1.5)
Highest educational level, n (%) ***			
Less than high/secondary education	38 (5.6)	152 (12.1)	190 (9.8)
Completed high/secondary school or technical college	436 (64.7)	849 (67.6)	1285 (66.6)
Completed university degree or professional education	200 (31.7)	254 (20.2)	454 (23.5)
***Perceived financial status, n (%)*** ^***^			
Poor/Very tight	238 (35.3)	396 (31.6)	634 (32.9)
Tight/Modestly comfortable	390 (57.9)	656 (52.3)	1046 (54.2)
Comfortable/Very comfortable	46 (6.8)	203 (16.2)	249 (12.9)
**Household income,** ***n*** **(%)** ^***^			
Less than $5,000 to less than $15,000	51 (7.6)	137 (10.9)	188 (9.7)
$15,000 to less than $50,000	219 (32.5)	544 (43.3)	763 (39.6)
$50,000 to less than $100,000	246 (36.5)	402 (32.0)	648 (33.6)
$100,000 and over	158 (23.4)	172 (13.7)	330 (17.1)
**Social support,** ***n*** (%) ***			
Low social support (0–2 persons)	8 (1.2)	38 (3.0)	46 (2.4)
Medium social support (3–4 Persons)	22 (3.3)	84 (6.7)	106 (5.5)
High Social support (≥5 Persons)	644 (95.5)	1133 (90.3)	1777 (92.1)
**Self-rated general health,** ***M(SD)*** ***	3.5 (1.0)	2.8 (1.0)	3.0 (1.0)
**Social vulnerability index,** ***n*** (%) **			
≥ 2 social vulnerability indicators	19 (2.8)	79 (6.3)	98 (5.1)
How often a family doctor or nurse explores how manageable treatments would be for you, M(SD)*	3.3 (0.8)	3.2 (0.8)	3.3 (0.8)
In the last 12 months, has the healthcare team here provided everything you need to help you manage your health concerns, *M(SD)*	4.5 (0.8)	4.4 (0.9)	4.5 (0.8)
Does the healthcare team here help you feel that your everyday activities such as diet and lifestyle make a difference to your health, *M(SD)*	3.16 (0.9)	3.2 (0.9)	3.2 (0.9)
Overall self-efficacy score, *M(SD)* ^***^	7.47 (1.41)	6.84 (2.04)	7.06 (1.88)
Quality of life (HUI) index, Mdn (IQR)***	0.91 (0.18–0.95)	0.809 (0.20–0.95)	0.85 (0.012–0.95)

*Note*: *N*: number of cases; %: Percent; *M*: Mean: *SD* (Standard deviation); *Mdn*: Median; *IQR*: Interquartile range; +**Ethno-cultural groups: Other include** Arab, Black, Chinese, Filipino, Japanese, Latin American, South East, and Asian West Asian. Missing data ranged from: 0.0% to 3.6%. Transgender, transsexual or a history of transitioning had few cases (*n* = 5, 0.3%), therefore this category was not included in the analysis. **Another language:** Arab, Spanish, Germany; HUI: Health utility index. Missing data for self-efficacy ranged from 3.7% to 4.4%. The score for self-efficacy ranged from 1 to 10 while range for health utility index ranged from: 0.012 to 0.95). Higher score indicates higher self-efficacy for managing chronic disease. Higher HUI indicates better quality of life. *: p-value < .05; **: *p* < .01; ***: *p* < .001. Statistical-tests (chi-square test for categorical and t-test for continuous variables, and Mann-Whitney test for quality of life variable). Higher score indicates better experience with help from the healthcare team.

### Self-reported patient experiences with help from the healthcare team, self-efficacy and quality of life

Table 1 shows results of self-reported patient experiences in primary care. Participants mostly appreciated how often a family doctor or a nurse explored the manageability of treatment for them (*M* = 3.3; *SD* = 0.8) and appreciated the help received from the health care team to manage their health concerns (*M* = 4.5; *SD* = 0.8). Additionally, participants reported good experiences with help they received from the health care team to improve their health through everyday activities such as diet and lifestyle (*M* = 3.2; *SD* = 0.9). Participants ’ SEMCD ranged from 1 to 10 (*M* = 7.06; *SD* = 1.88) while quality of life ranged from 0.02 to 0.95 (*Mdn* =0.85) (Table 1).

### Practice characteristics

Table 2 provides characteristics of all participating practices. Eighty-seven practices across three regions participated in the study. For all 87 practices, more than a third had RNs in their clinics. In addition to physicians, other health professionals in the practices included social workers, psychologists, nutritionists, nurse practitioners, pharmacists, occupational therapists, physiotherapists, etc. More than half had large team size (≥11 members) and the majority of practices (60%) were in fee-for-services models. Practice characteristics did not differ among those with and without sufficient TCI data.

**Table 2.** Practice characteristics

**Variable, *n* (%)**	**Practices with sufficient TCI data** ***n*** **= 63**	**Practices without sufficient TCI data** ***n*** **= 24**	**All participating practices** ***N*** **= 87**
**Presence of RNs in the clinic**			
Yes	25 (39.7)	5 (20.8)	30 (34.5)
No	38 (60.3)	19 (79.2)	57 (65.5)
**Team size**			
≤5	14 (22.2)	8 (34.8)	22 (25.6)
6–10	13 (20.6)	5 (21.7)	18 (20.9)
≥11	36 (57.1)	10 (43.5)	46 (53.5)
**Physician’s payment model**			
Fee-for-services	36 (57.1)	14 (66.7)	50 (59.5)
Capitation or roster	14 (22.2)	3 (14.3)	17 (20.2)
Salary or (hourly rate, sessional payment, contract)	8 (12.7)	1 (4.8)	9 (10.7)
Blended model (mix of different payment models)	2 (3.2)	1 (4.8)	3 (3.6)
Other	3 (4.8)	2 (9.5)	5 (6.0)

*Note*: Missing data ranged from 0.0 % to 3.7%. Statistical tests (chi-square test for categorical variable and t-test for continuous variable); *: *p* < .05; **; *p* < .01; *** *p* < .001.

Table 3 indicates practices with and without sufficient TCI data. Sixty-three (72%) practices had sufficient TCI data. The average score of TCI was 72.74 (*SD* = 10.8) on a scale of 0–100, suggesting high team functioning but with room for improvement. Results of bivariate analyses can be found in the supplemental materials Table 4S and Table 5S.

**Table 3.** Individual and overall TCI scores

**Team functioning (TCI score),** ***M (SD)***	**Practices with TCI valid data *n* = 63**
TCI_Participative safety	75.35 (11.4)
TCI_Supportive new ideas and innovation	71.79 (12.2)
TCI_objectives	72.00 (14.5)
TCI_orientation	71.20 (11.8)
TCI_overall	72.74 (10.8)
Min - Max	39–98

*Note*: A total of 63 practices had sufficient TCI data, therefore 24 (27.6%) practices without sufficient TCI data were removed from the analysis. A higher score of TCI score indicates increased/higher team functioning. Min: Minimum; Max: Maximum.

### The association between team functioning and self-efficacy for managing chronic conditions

**Figure 1.**
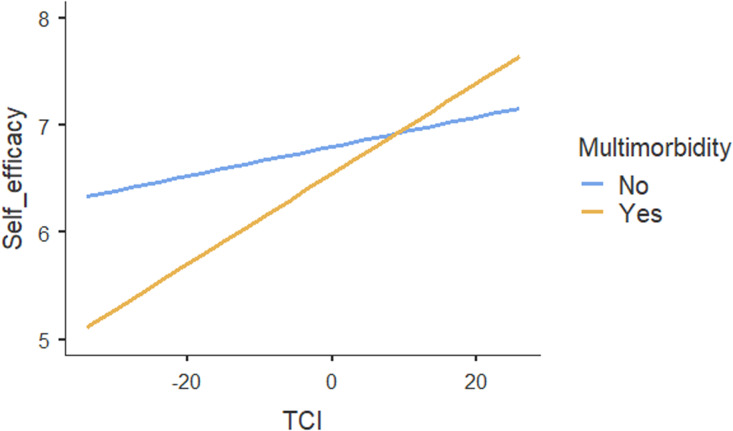
Moderating effect of multimorbidity on the association between team functioning and self-efficacy for managing chronic conditions.

Table 4 shows the results of regression analyses. The ICC for the two-level mixed-random linear regression model was .05, indicating that 5% of variations in patients’ self-efficacy could be explained by the practices. Both within-group variance (*p* < .001) and between-group variance (*p* = .02) in self-efficacy scores were statistically significant, suggesting that patients’ self-efficacy differed between and within practices. The regression results showed no association between TCI and self-efficacy. Moreover, social vulnerability had no influence on the association between TCI and patients’ self-efficacy. In contrast, our results suggested a significant association between TCI and self-efficacy ***(p < .01)*** for patients with multimorbidity compared with no multimorbidity (Figure 1). Age, self-rated health and patients’ experiences on provision of everything needed to help patients manage their health concerns were also positively associated with self-efficacy. The increase of one unit in these variables, self-efficacy increased by .01, .94 and .19 respectively. Other individual patient and practice characteristics were not associated with self-efficacy.

**Table 4.** Association between team functioning and outcomes: results of regression analyses

**Variables**	**Self-efficacy**			**Quality of life**		
	***B***	**95%CI of *B***			**95%CI of *B***	
		**Lower**	**Upper**	***B***	**Lower**	**Upper**
Fixed effects						
Intercept	3.70	2.02	.37***	−.63	−.86	−.41***
Team functioning	−.01	−.03	.01	.00	−.00	.00
Age	.01	.00	.02**	−.00	−.00	.00
Sex: Male_ref						
Sex: Female	−.09	−.30	.13	.00	−.03	.04
Household income: Less than $5,000 to less than $15,000_Ref						
Household income: $15,000 to less than $50,000	.03	−.33	.40	.04	−.03	.11
Household income: $50,000 to less than $100,000	.23	−.15	.60	.07	.01	.13*
Household income: $100,000 and over	.29	−.14	.71	.09	.02	.15**
Social vulnerability index: 0 or 1 SV_indicator_Ref						
≥ 2 SV-indicators	−3.84	−7.77	.09	−2.42	−3.54	−1.29***
Multimorbidity_No_Ref						
Multimorbidity-Yes	−2.34	−3.80	−.87**	−.14	−.30	.02
Self-rated general health	.94	.84	1.05	.12	.11	.14***
How often a family doctor or nurse explores how manageable treatments would be for you	−.07	−.21	.07	−.02	−.03	.00
In the last 12 months, has the healthcare team here provided everything you need to help you manage your health concerns	.19	.06	.32**	.02	−.00	.04
*Does the healthcare team here help you feel that your everyday activities such as diet and lifestyle make a difference to your health?*	.03	−.10	.16	−.01	−.03	.01
Presence of a RN in the clinic_No_Ref						
Presence of the RN in the clinic, yes	.03	−.34	.40	−.01	−.05	.03
Physician’s payment model_fee-for-services_Ref						
Physician’s payment model: Blended model (mix of different payment models)	−.07	−.85	.72	.02	−.01	.05
Physician’s payment model: Salary or (hourly rate, sessional payment, contract)	−.06	−.52	.40	.03	−.01	.06
Physician’s payment model: Capitation or roster	.03	−.35	.42	.01	−.04	.05
Physician’s payment model: Others	.21	−.49	.90	.04	−.03	.12
Team size: ≤ 5 members_Ref						
Team size: 6–10 members	−.07	−.53	.39	−.01	−.06	.05
Team size: ≥ 11 members	−.19	−.62	.24	−.02	−.08	.04
TCI*<2 social vulnerabilities_Ref						
TCI*≥ 2 social vulnerabilities	.04	−.01	.09	.03	.02	.04***
TCI*Multimorbidity_No_Ref						
TCI*Multimorbidity_Yes	.03	.01	.05**	.00	−.00	.00
**Random effects**						
Within-group variance	2.53	2.32	.77***	.06	.05	.06***
Between-group variance	.13	.06	.30**	.00		

*Note:* **TCI:** Team Climate Inventory; Sex (1 = Female; 2 = Male); household income (1 = Less than $5,000 to less than $15,000; 2 = $15,000 to less than $50,000; 3 = $50,000 to less than $100,000; 4 = $100,000 and over); Social vulnerability indicators (1 = 0 or 1 social vulnerability indicator; 0 = ≥ 2 social vulnerabilities); multimorbidity (1 = No; 0 = Yes); Self-rated general health (1 = poor; 2 = fair; 3 = Good; 4 = Very good; 5 = Excellent); How often a family doctor or nurse explores how manageable treatments would be for you (1 = Not at all; 2 = Little; 3 = mostly; 4 = completely; 99 = N/A); in the last 12 months, has the healthcare team here provided everything you need to help you manage your health concerns (1 = No, not at all; 2 = No, not really; 3 = Yes, to some extent; 4 = Yes, mostly; 5 = Yes, definitely; 99 = N/A: No, I did not need such support); Does the healthcare team here help you feel that your everyday activities such as diet and lifestyle make a difference to your health (1 = No, not at all; 2 = No, not really; 3 = Yes, to some extent; 4 = Yes, definitely); registered nurses (1 = No; 0= Yes); Physician’s payment model (4 = fee-for-services; 3 = blended model; 2 = salary or hourly rate, sessional payment model, contract; 1 = capitation or roster; 0 = others); team size (3 = ≤ 5 members; 2 = 6–10 members; 1 = ≥ 11 members); df: degree freedom; *: *p* < .05; **: *p* < .01; ***: *p* < .001.

### The association between team functioning and quality of life

**Figure 2.**
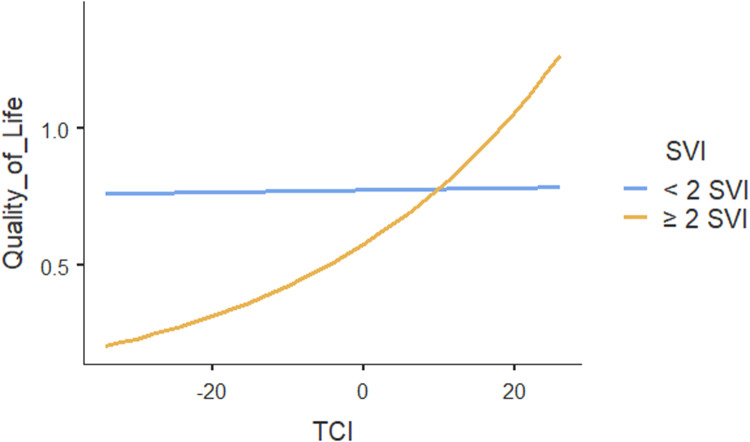
Moderating effect of social vulnerability index (SVI) on the association between team functioning and quality of life.

The ICC for the two-level mixed-random effects model was 0.00, indicating no variation in patients’ quality of life could be explained by the practices. Only the within-group variance component in quality of life was statistically significant (*p* < .001), suggesting that patients’ quality of life differed within practices. Table 4 shows the results of regression analysis which also showed no association between team functioning and patients’ quality of life (*p* = .95). However, team functioning had significant positive association with quality of life (***p*** **< .001**) for patients with two or more social vulnerabilities (Figure 2). Our results showed that multimorbidity does not influence the association between team functioning and quality of life. Other patient characteristics including household income and self-rated general health were positively associated with quality of life. None of the other individual or practice characteristics were associated with the quality of life.

## Discussion

This study examined the direct association between primary care team functioning and patients’ SEMCD and quality of life. We also examined the moderating effect of social vulnerability and multimorbidity on this association. To our knowledge, this is the largest Canadian study about team functioning in primary care. A unique strength of this study is its equity-focused approach, examining how the impact of team functioning may differ based on patients’ social vulnerability and multimorbidity status.

The team functioning score in our study was similar to other studies that were conducted in Canadian primary care practices using TCI scale (Howard *et al.*, 2011; Beaulieu *et al.*, 2014), with some primary care teams having very low team functioning scores while others demonstrating higher scores of team functioning. Different levels of team functioning scores across practices highlight that primary care team is context-specific (Bates *et al.*, 2025), with some teams still facing challenges to work effectively while others have advanced in effective working as primary care teams. Health care leaders and primary care practitioners should set strategies to support teams that are struggling and sustain teams that are already functioning effectively. Ensuring regular team meetings through informal and formal interactions, conflict resolutions, addressing difficulties in information sharing between team members, ensuring participation of all team members in clinical decision-making and care coordination could enhance team functioning (Xyrichis and Lowton, 2008; Valaitis *et al*., 2020; Salas *et al.*, 2017).

Lack of an association between primary care team functioning and patients’ self-efficacy or quality of life is consistent with previous studies which did not find an association between team functioning and self-efficacy or quality of life (Becker and Roblin, 2008; Schuttner *et al.*, 2020), and other outcomes such as quality of care among primary care patients in the Netherlands and the UK (Hann *et al.*, 2007; Bosch *et al.*, 2008; Goh *et al.*, 2009) or quality of care indicators [e.g., HbA1C, total cholesterol, systolic blood pressure and process of quality of diabetes care] (Hann *et al.*, 2007; Bosch *et al.*, 2008; Goh *et al.*, 2009). The lack of association may be partly attributed to the methodological approach used in this study, particularly the characteristics of the population and the types of outcomes examined, which can influence the ability to detect an association (Goh *et al*., 2009).

General primary care patients reported relatively high levels of self-efficacy and quality of life, leaving limited room for observable influence. The range of quality of life score mirrors the general Canadian population (Xie *et al*., 2016). In contrast, patients with multimorbidity and those experiencing greater social vulnerabilities had lower scores, suggesting greater potential for observable effect. The general primary care patient population encompasses individuals with varying levels of complexity in their health care needs. Therefore, not all primary care patients require highly coordinated primary care services delivered by multiple health professionals (Langton *et al.*, 2020). Many are adequately supported through episodic care (single encounter) or preventive services (Langton *et al.*, 2020), which can be easily delivered by a single clinician (Langton *et al.*, 2020). Therefore, high levels of team functioning may not yield additional benefits for this broader patient group in terms of SEMCD and overall quality of life. It is essential for primary care practitioners to identifying which patients would most benefit from TBPC so that teams can better meet the healthcare needs of those who require more assistance in managing the complexities of their health.

Using an equity lens, this study reveals an important finding: high performing teams do not benefit all primary care patients equally, rather their impact is concentrated among specific population groups and suggests in which populations they matter the most. Our study findings suggested that team functioning has the most beneficial effect for those living with increased social vulnerabilities and multimorbidity. The association between team functioning and quality of life for individuals with increased social vulnerability was also clinically important since it is within the range of a minimal clinically important difference value of 0.02–0.04 HUI (Horsman *et al.*, 2003). That is, high team functioning is beneficial in improving the quality of life for patients with two or more social vulnerabilities. High team functioning could be needed for well coordinated health care services from multiple professionals (Makovski *et al.*, 2019) to effectively meet the care needs of people with more social vulnerabilities.

Usually, health professionals from different disciplines have different priorities and objectives which hinder teams’ ability to provide adequate support to patients (Cramm *et al.*, 2014). Hence, teams with shared objectives and committed to high-quality of care and support from each other are better able to address these challenges by providing care coordination and are better able to provide required resources and, medical and psychosocial support which are important for people with more social vulnerabilities (Cramm *et al.*, 2014). Therefore, a high functioning team could effectively connect and link patients to specific social determinants of health-related social services and community resources (Whitman *et al.*, 2022).

For example, teams could connect equity deserving patients to the right provider who can allow them sufficient time to express their emotions and feelings, resulting in improved quality of life. Our findings suggest that well-functioning teams can enable all health care providers, such as physicians, nurses, nurse practitioners, pharmacists, etc., to focus on complex patients, particularly those with multimorbidity or social vulnerabilities. More work is needed in understanding how best to deploy the talents and expertise of individual team members, who have a diversity of discipline-specific training. Screening primary care patients for social vulnerabilities and complexity of care needs can help practitioners identify who require additional resources and guide strategies for effective use and coordination of resources, and providing appropriate care to patients. Pre-visit questionnaires and automatic electronic medical records or automatic information technology-based tools can be employed to flag patients with social vulnerabilities and or multimorbidity (Wagner *et al*., 2017).

Additionally, to nurture effective team functioning and ensure intended patient outcomes are achieved, all primary care practitioners/team members should safely participate in clinical decision-making and use their full scope of practice (Zajac *et al*., Zajac *et al*., 2021; Zhang, 2024). Therefore, policymakers should develop health policies that create safe and enabling environments for primary care practitioners to fully enact their legal scope of practice (Zhang, 2024). Health policies that eliminate financial and regulatory barriers preventing practitioners (e.g., registered nurses, pharmacists) from fully exercising their roles can facilitate primary care team functioning, enable meaningful contributions to patient care, and ultimately improve patient outcomes. Enabling health care providers to care for medically *and* socially complex patients would improve patient outcomes and begin to address health disparities in primary care patients (Cody *et al.*, 2020).

Our findings are consistent with qualitative studies showing that TBPC improved confidence, knowledge, skills, motivation and self-efficacy for people managing their chronic conditions for those with multimorbidity (Ngangue *et al.*, 2020; Sasseville *et al.*, 2021). These qualitative studies accredited effectiveness of team-based care on attributes of team functioning where there is regular collaboration, communication and information exchange (Ngangue *et al.*, 2020). Teams with these attributes can provide better supports and comprehensive services to patients with complex care needs (Frogner *et al.*, 2018; Langton *et al.*, 2020).

Consistent with previous literature, older age, self-rated health and patient-reported experience with care were positively associated with self-efficacy (Riazi *et al.*, 2004; Alegría *et al.*, 2009; Lemieux *et al.*, 2011; Loeb *et al.*, 2011; Kimerling *et al.*, 2016; Rutten *et al.*, 2016; Chan *et al.*, 2021; Melin *et al.*, 2023).

None of practice characteristics (presence of RN in the clinic, physician’s payment model and team size) were associated with patients’ self-efficacy. Two potential reasons could explain these findings. First, it is possible that the presence of RNs, physician’s payment model and team size are only indirectly associated with patient’s self-efficacy through enabling primary care providers with the ability to provide comprehensive self-management support (Lemieux *et al.*, 2011). Second, practice characteristics are likely too distal from the outcome variables of interest. There is likely a more complex pathway that practice characteristics influence patient’s self-efficacy and quality of life which should be explored in future work.

## Limitations

This was a cross-sectional study where no causation can be inferred. The results are still relevant given the infancy of team-based care in the Canadian primary care system (Khan *et al.*, 2021) and new team-based care initiatives still being established in primary care practices (Sibbald *et al.*, 2021). Additionally, this study used data collected from 2014–2016 before the COVID-19 pandemic. Team functioning in primary care may look markedly different in the post pandemic context, as substantial policy changes including the rapid expansion of virtual care have reshaped how healthcare services are organized and delivered and may have influenced the implementation of team-based care in primary care. While one of the largest studies on team functioning, it used data from only a small number of primary care practices across Canada. Therefore, the results are not generalizable to all the primary care practices in Canada. Future studies could use these baseline findings for further large-scale studies to examine the effect of team functioning in primary care in Canada. In addition, future research using a longitudinal design should examine how team functioning changes over time and how the change affects patient’s self-efficacy and quality of life as well as how to sustain optimal team functioning in primary care practices.

## Conclusion

Given the increase of patients with chronic conditions and multimorbidity as well as patients with complex social needs in primary care, examining the association between team functioning and patients’ SEMCD and quality of life in primary care is a subject of high interest for clinicians and policy perspectives. The study findings provide insights on the strong positive association between primary care team functioning and self-efficacy for PLWMM. Those with increased social vulnerabilities report increased quality of life from higher functioning teams. The study findings make an important contribution to the primary care team literature as they indicate the value of high team functioning in improving health equity in primary care; and the clinical benefit of team functioning for primary care patients. Based on the positive value of team functioning on patient outcomes, primary care clinicians and policy makers should avail resources and strategies to support effective team functioning in primary care.

## Supporting information

Ndateba et al. supplementary material Ndateba et al. supplementary material

## Data Availability

All data used in this study are deposited in the University of British Columbia secure system and can be available upon reasonable request from Sabrina Wong. Send an email request to: Email: sabrina.wong@ubc.ca

## Supplementary material

The supplementary material for this article can be found at https://doi.org/10.1017/S1463423626100887

## Previous presentation

The 36^th^ Annual Health Policy Conference: Poster Presentation, March 2024.
