# Supplementary material for: Association between team functioning and self-efficacy and quality of life for primary care patients in British Columbia, Nova Scotia, and Ontario
Source: Prim Health Care Res Dev. 2026 Feb 12;27:e20. doi: 10.1017/S1463423626100887 (PMC12931995; doi:10.1017/S1463423626100887)
Supplement: Ndateba et al. supplementary material [file S1463423626100887sup001.docx]

**Supplemental materials**

**Table 1S: Patients**

|  | **Variable** | **Attribute** |
| --- | --- | --- |
| 1 | Age: What is your date of birth? Year |  |
| 2 | Sex | 1=Male 2=Female |
| 3 | Educational level  Which of the following best describes the highest level of education you have completed? | 1=Did not complete secondary school or high school 2=Completed secondary school or high school 3=Had some university education or completed a community college, technical college, or postsecondary program (*e.g., trade, technical or vocational school, CEGEP*) 4=Completed a bachelor’s degree (*e.g., B.A., B.Sc., B.S.N.*) 5=Completed a graduate or professional degree (*e.g., MD, DDS, DMD, DVM, OD, PhD*) |
| 4 | Marital status | 1=Married or living with a partner 2=Separated  3=Divorced 4=Widowed 5=Never married |
| 5 | Perceived financial status | 1=Poor 2=Very tight  3=Tight 4=Modestly comfortable 5=Comfortable 6=Very comfortable |
| 6 | Household income | 01=Less than $5,000 02=$5,000 to less than $10,000 03=$10,000 to less than $15,000 04=$15,000 to less than $20,000 05=$20,000 to less than $30,000 06=$30,000 to less than $40,000 07=$40,000 to less than $50,000 08=$50,000 to less than $60,000 09=$60,000 to less than $70,000 10=$70,000 to less than $80,000 11=$80,000 to less than $90,000 12=$90,000 to less than $100,000 13=$100,000 to less than $150,000 14=$150,000 and over |
| 7 | Immigration status: Born in Canada | 1=No  2= Yes |
| 8 | Immigration:  What year did you first come to Canada to live? |  |
| 9 | Number of chronic diseases |  |
| 10 | **Social support** |  |
| 11 | If you needed it, how many persons, family or friends, could help you with activities of daily living (e.g., dressing, driving)? (Social support tangible) | 1=None 2=One 3=More than one |
| 12 | How many persons, family or friends, show you love and affection when you need it? (Social support emotional) | 1=None 2=One 3=More than one |
| 13 | With how many persons, family or friends, can you freely share good time with? (Social support emotional) | 1=None 2=One 3=More than one |
| 14 | With how many persons, family or friends, can you freely confide in or talk about yourself or your problems? Social support emotional) | 1=None 2=One 3=More than one |

**Supplemental materials**

**Table 2S: Items for TCI**

| **Items** | **Strongly disagree**  **1** | **Disagree**  **2** | **Neither agree nor disagree**  **3** | **Agree**  **4** | **Strongly agree**  **5** |
| --- | --- | --- | --- | --- | --- |
| **Part 1: Participation in the Team. To what extent do you agree with the following?** | | | | | |
| We have a "we are in it together" attitude |  |  |  |  |  |
| People keep each other informed about work-related issues in the team |  |  |  |  |  |
| People feel understood and accepted by each other |  |  |  |  |  |
| There are real attempts to share information throughout the team |  |  |  |  |  |
| There is a lot of give and take |  |  |  |  |  |
| We keep in touch with each other as a team |  |  |  |  |  |
| **Part 2: Support for New Ideas: To what extent do you agree with the following?** | | | | | |
| This team is always moving towards the development of new answers |  |  |  |  |  |
| This team is open and responsive to change |  |  |  |  |  |
| People in this team are always searching for fresh, new ways of looking at problems |  |  |  |  |  |
| Members of the team provide and share resources to help int he application of new ideas |  |  |  |  |  |
| Team members provide practical support for new ideas and their application |  |  |  |  |  |

| **Part 3: Team Objectives:**  **Select the appropriate number to indicate how far each statement describes your team.** | **1 Not at all** | **2** | **3** | **4 Somewhat** | **5** | **6** | **7 Completely** |
| --- | --- | --- | --- | --- | --- | --- | --- |
| How clear are you about what your team's objectives are? |  |  |  |  |  |  |  |
| How far are you in agreement with these objectives? |  |  |  |  |  |  |  |
| To what extent do you think other team members agree with these objectives? |  |  |  |  |  |  |  |
| To what extent do you think these members of your team are committed to these objectives? |  |  |  |  |  |  |  |

| **Part 4: Task Orientation**  select the response which you think best describes your team. | **1 To a very little extent** | **2** | **3** | **4 To some extent** | **5** | **6** | **7 To a very great extent** |
| --- | --- | --- | --- | --- | --- | --- | --- |
| Do your team colleagues provide useful, ideas and practical help to enable you to do the job to the best of your ability? |  |  |  |  |  |  |  |
| Are team members prepared to question the basis of what the team is doing? |  |  |  |  |  |  |  |
| Does the team critically appraise potential weaknesses in what itis doing in order to achieve the best possible outcome? |  |  |  |  |  |  |  |
| Do members of the team build on each other's ideas in order to achieve the best possible outcome? |  |  |  |  |  |  |  |

**Supplemental materials**

**Table 3S: Items for self-efficacy for managing chronic conditions**

| 1 | How confident are you that you can keep the fatigue caused by your health condition from interfering with the things you want to do? | 01=Not confident at all  02= 03= 04= 05= 06= 07= 08= 09= 10=Totally confident 99=NA |
| --- | --- | --- |
| 2 | How confident are you that you can keep the physical discomfort or pain of your health condition from interfering with the things you want to do? | 01=Not confident at all 02= 03= 04= 05= 06= 07= 08= 09= 10=Totally confident 99=NA |
| 3 | How confident are you that you can keep the emotional distress caused by your health condition from interfering with the things you want to do? | 01=Not confident at all 02= 03= 04= 05= 06= 07= 08= 09= 10=Totally confident 99=NA |
| 4 | How confident are you that you can keep any other symptoms or health problems you have from interfering with things you want to do? | 01=Not confident at all 02= 03= 04= 05= 06= 07= 08= 09= 10=Totally confident 99=NA |
| 5 | How confident are you that you can do the different tasks and activities needed to manage your health condition so as to reduce your need to see a doctor? | 01=Not confident at all 02= 03= 04= 05= 06= 07= 08= 09= 10=Totally confident 99=NA |
| 6 | How confident are you that you can do things other than just taking medication to reduce how much your illness affects your everyday life? | 01=Not confident at all 02= 03= 04= 05= 06= 07= 08= 09= 10=Totally confident 99=NA |
| **SECDM score** | Self-efficacy for managing chronic diseases scale constructed | 1,2,3,4,5,6 |

The score for self-efficacy ranged from 1 to 10. Higher score indicates higher self-efficacy for managing chronic disease.

**Table 4S: Bivariate association of self-reported patient characteristics, self-reported experiences with help from the health care team and practice characteristics with Quality of life**

| **Variables** | **EQ-5D-5L** | | |
| --- | --- | --- | --- |
|  | **N** | **M*dn (IQR)*** | **Mann Whitney/ Kruskal-Wallis test /Spearman’s rho** |
| **Age** | 1929 |  | **-.104^e***^** |
| ***Sex***  Male  Female | 638  1285 | 0.86(0.20-0.95)  0.85(0.21-.95) | 395713^f^ |
| **Household income**  Less than $5,000 to less than $15,000 $15,000 to less than $50,000 $50,000 to less than $100,000 $100,000 and over | 188  763  648  330 | 0.79(0.21-.95)  0.83 (0.21-.95)  0.86(0.22-.95)  0.87(0.20-.95) | **96.264^g***^** |
| **≥ 2 social vulnerability indicators**  No  Yes | 1831  98 | 0.85(0.20-0.95)  0.74(0.21-0.95) | **55718^f***^** |
| **Multimorbidity**  No  Yes | 674  1255 | 0.91(0.22-0.95)  0.82(0.20-0.95) | **226697^f***^** |
| **Self-rated general health** | 1872 |  | **.606^e^***** |
| How often a family doctor or nurse explores how manageable treatments would be for you? | 1534 |  | .026 |
| In the last 12 months, has the healthcare team here provided everything you need to help you manage your health concerns? | 1792 |  | .**143**** |
| Does the healthcare team here help you feel that your everyday activities such as diet and lifestyle make a difference to your health? | 1861 |  | .039 |
| **Presence of RNs in the clinic**  No  Yes | 1236  693 | 0.85(0.20 - 0.95)  0.84(0.21 - 0.95) | 413585^f^ |
| **Team size**  ≤ 5  6-10  >=11 | 473  396  1039 | 0.86(0.21 - 0.95)  0.85(0.21- 0.95)  0.85(0.20-0.95) | 1.184^g^ |
| **Physician’s payment model**  Fee-for-services  Capitation or roster  Salary or (hourly rate, sessional payment, contract)  Blended model (mix of different payment models) Other | 1115  364  196  65  117 | 0.85(0.20-0.95)  0.87(0.21-0.95)  0.83(0.22-0.95)  0.83(0.23-0.95)  0.85(0.26-0.95) | 7.805^g^ |
| **Team functioning (TCI-score)** | 1416 |  | .**085^e**^** |

***Note: ^e^:*** *Spearman’s rho correlation;* ***^f^:*** *Mann Whitney U test****; ^g^:*** *Kruskal-Wallis-Test;* ***Mdn:*** *Median****; IQR:*** *Interquartile range****. *: p<.05; **: p<.01; ***: p<.001***

**Table 5S:** **Bivariate association between self-reported patient characteristics, patient experiences with help from the health care team and practice characteristics with self-efficacy**

| **Variables** | **Self-efficacy** | | | |
| --- | --- | --- | --- | --- |
|  | **N** | **M*(SD)*** | **95%CI** | ***F/t/ r*** |
| **Age** | 1929 |  |  | .**061^a^**** |
| ***Sex***  Male  Female | 638  1286 | 7.23(1.85)  6.98(1.88) | 7.08-7.37  6.87-7.08 | **2.775^b^**** |
| **Household income**  Less than $5,000 to less than $15,000 $15,000 to less than $50,000 $50,000 to less than $100,000 $100,000 and over | 188  763  648  330 | 6.19(2.27)  6.89(1.93)  7.33(1.65)  7.39(1.68) | 5.87-6.52  6.76-7.03  7.20-7.45  7.21-7.57 | **24.043^c^***** |
| **≥ 2 social vulnerability indicators**  No  Yes | 1831  98 | 7.12 (1.83)  5.85 (2.19) | 7.04-7.21  5.41-6.29 | **5.647^b^**** |
| **Multimorbidity**  No  Yes | 674  1255 | 7.47(1.41)  6.84(2.04) | 7.36-7.57  6.72-6.95 | **7.947^b^***** |
| Self-rated general health | 1872 |  |  | **.493^a^**** |
| How often a family doctor or nurse explores how manageable treatments would be for you? | 1534 |  |  | **.091^a^**** |
| In the last 12 months, has the healthcare team here provided everything you need to help you manage your health concerns? | 1792 |  |  | **.189^a^**** |
| Does the healthcare team here help you feel that your everyday activities such as diet and lifestyle make a difference to your health? | 1861 |  |  | **.104^a^**** |
| **Presence of RNs in the clinic**  No  Yes | 1236  693 | 7.10(1.84)  6.98(1.93) | 7.00-7.20  6.83-7.12 | .157^b^ |
| **Team size**  ≤ 5  6-10  >=11 | 473  396  1039 | 7.22(1.85)  7.05(1.82)  6.98(1.89) | 7.05-7.39  6.87-7.23  6.87-7.10 | 2.665^c^ |
| **Type of physician’s remuneration**  Fee-for-services  Capitation or roster  Salary or (hourly rate, sessional payment, contract)  Blended model (mix of different payment models) Other | 1115  364  196  65  117 | 7.07(1.85)  7.27(1.77)  6.70(1.91)  6.76(2.31)  7.12(2.05) | 6.96-7.18  7.08-7.44  6.43-6.97  6.18(7.33)  6.75-7.50 | **3.305^c^*** |
| **Team functioning (TCI-score)** | 1416 |  |  | **.078^a^**** |

***Note***: M: Mean; SD: Standard deviation; CI: Confidence interval; ^a^: Pearson’rho correlation (*r*); ^b^: *t* (T-test); ^c^: F (One-Way ANOVA test); **: p <.05; **: p<.01; ***: p<.001*

**Appendix A: A list of chronic disease**

- Hypertension
- Depression
- Chronic musculoskeletal conditions causing pain,
- Osteoarthritis and other arthritis
- Osteoporosis
- Chronic obstructive pulmonary diseases and asthma or bronchitis,
- Stomach problems,
- Colon problems (chronic inflammatory disease irritable bowel syndrome)
- Chronic liver disease
- Diabetes
- Thyroid disorder
- Cancer (but not skin cancer)
- Obesity
- Chronic kidney disease or failure
- Cholesterol problem or hyperlipidemia
- Cardiovascular diseases (e.g., angina, previous myocardial infarction)
- Atrial fibrillation
- Lower limb circulation
- Chronic urinary problem
- Heart failure (heart valve replacement)
- Stroke and transient ischemic attacks
- Dementia or Alzheimer’s
- Others (specify)

**Appendix B: Control variables**

| **Potential confounding variables** | **Variable** | **Responses** |
| --- | --- | --- |
| **Organizational characteristics (n=3)** | Team size | 1= ≥ 11 team members  2= 6-10 team members  3= ≤ 5 team members |
|  | Presence of registered nurses in the clinic | 1= No  0= Yes |
|  | Physician’s payment model | 0= Other  1= Capitation or roster  2= Salary or (hourly rate, sessional payment, contract) models)  3= Blended model (mix of different payment models)  4= Fee-for-services |
| **Individual patient characteristics (n=7)** | Age | Years |
|  | Sex | 1= Female  2= Male |
|  | Household income | 1= $5000 to < $15000  2= $15000 to < $50000  3= $50000 to < $100000  4= $100000 and over |
|  | Self-rated general health | 1= Poor  2= Fair  3= Good  4= Very good  5= Excellent |
|  | Ethno-cultural groups | 1= Caucasian (of European descent)  2= South Asian  3= Other |
|  | Social support | 1= Low social support (0- 2 persons available for providing social support)  2= Medium social support (3-4 persons)  3= High social support (5+ persons) |
| **Patient-reported experiences with help from the healthcare team (n=3)** | How often a family doctor or nurse explores how manageable treatments would be for you? | 1= Not at all  2= Little  3= Mostly  4= Completely  99= No treatments have been prescribed (N/A) |
|  | In the last 12 months, has the healthcare team here provided everything you need to help you manage your health concerns? | 1= No, not at all  2=No, not really  3=Yes, to some extent  4= Yes, mostly  5= Yes, definitely  99= No, I did not need such support (N/A) |
|  | Does the healthcare team here help you feel that your everyday activities such as diet and lifestyle make a difference to your health? | 1=No, not at all  2=No, not really  3=Yes, to some extent  4= Yes, definitely |

***Note***: **Ethno-cultural groups include:** Caucasians (European descents)**;** South Asian (East Indian, Pakistani, Sri Lankan) and other**:** Arab**,** Black**,** Chinese, Filipino**,** Japanese, Latin American, South East, and Asian West Asian.
